# Assessment and Mitigation of Caregiver Burden in a Movement Disorder Clinic

**DOI:** 10.1093/geroni/igaf122.797

**Published:** 2025-12-31

**Authors:** Morgan Wilhelmi, Joseph Quinn, Shannon Anderson, Rachel Lynn, Jiayun Xu

**Affiliations:** Oregon Health & Science University, Portland, Oregon, United States; Oregon Health & Science University, Portland, Oregon, United States; Oregon Health & Science University, Portland, Oregon, United States; Oregon Health & Science University, Portland, Oregon, United States; Oregon Health & Science University, Portland, Oregon, United States

## Abstract

Finding ways to assess and mitigate caregiver burden in real life clinical settings is a challenge. Using a pre-post test design in a quality improvement project, we aimed to assess the feasibility and preliminary efficacy of a social work intervention to reduce caregiver burden. From June 2023 to May 2024, caregiver burden was assessed using the Zarit Caregiver Burden Inventory on family caregivers who accompanied patients to a movement disorders clinic visit. Caregivers who scored above 30 on the Zarit were referred to a social worker who completed a structured needs assessment and resource referrals. The Zarit was measured again 2 months post social work referral. Burden was assessed among 405 caregivers at baseline, and only 3.5% (n = 14) caregivers completed a two-week assessment. Out of the caregivers who reported higher burden (n = 106, 30.1%), 69.8% (n = 74) were contacted by social work, and 44.3% (n = 47) completed a social work visit. The most common sources of caregiver burden were: mental health (n = 25, 29.8%), and financial concerns (25.5%). Of the caregivers who completed a 2-week Zarit, 42.9% (n = 6) reported an improvement in caregiver burden. The low follow up rate highlights the challenges of measuring post-intervention scores in a real-life clinic setting. Nevertheless, results indicate caregivers were willing to engage with social workers to discuss their burden and come up with a plan to address burden. Future projects need to more systematically assess burden post-intervention and determine ways to measure engagement with community resources after social work visits occur.

